# Twist-assisted optoelectronic phase control in two-dimensional (2D) Janus heterostructures

**DOI:** 10.1038/s41598-023-39993-8

**Published:** 2023-08-22

**Authors:** S. Kar, P. Kumari, M. Venkata Kamalakar, S. J. Ray

**Affiliations:** 1 Department of Physics, Indian Institute of Technology Patna, Bihta, 801103 India; 2 Department of Physics and Astronomy, Uppsala University, Box 516, 75120 Uppsala, Sweden

**Keywords:** Condensed-matter physics, Electronic properties and materials

## Abstract

Atomically thin two-dimensional (2D) Janus materials and their Van der Waals heterostructures (vdWHs) have emerged as a new class of intriguing semiconductor materials due to their versatile application in electronic and optoelectronic devices. Herein, We have invstigated most probable arrangements of different inhomogeneous heterostructures employing one layer of transition metal dichalcogenide, TMD (MoS_2_, WS_2_, MoSe_2_, and WSe_2_) piled on the top of Janus TMD (MoSeTe or WSeTe) and investigated their structural, electronic as well as optical properties through first-principles based calculations. After that, we applied twist engineering between the monolayers from 0$^{\circ } \rightarrow$ 60$^{\circ }$ twist angle, which delivers lattice reconstruction and improves the performance of the vdWHs due to interlayer coupling. The result reveals that all the proposed vdWHs are dynamically and thermodynamically stable. Some vdWHs such as MoS_2_/MoSeTe, WS_2_/WSeTe, MoS_2_/WSeTe, MoSe_2_/MoSeTe, and WS_2_/MoSeTe exhibit direct bandgap with type-II band alignment at some specific twist angle, which shows potential for future photovoltaic devices. Moreover, the electronic property and carrier mobility can be effectively tuned in the vdWHs compared to the respective monolayers. Furthermore, the visible optical absorption of all the Janus vdWHs at $\theta$ = 0$^{\circ }$ can be significantly enhanced due to the weak inter-layer coupling and redistribution of the charges. Therefore, the interlayer twisting not only provides an opportunity to observe new exciting properties but also gives a novel route to modulate the electronic and optoelectronic properties of the heterostructure for practical applications.

## Introduction

Tremendous research attention is currently being focused on two-dimensional (2D) nanomaterials and their heterostructures due to their rich physical properties and diverse technological applications^1–3^. The transition metal dichalcogenides (TMDs) such as $\hbox {MoS}_{2}$, $\hbox {WS}_{2}$, $\hbox {MoSe}_{2}$, and $\hbox {WSe}_{2}$ are the most intriguing members of the 2D family due to their excellent electronic, thermal, and optical properties^4^. In addition to having graphene-like structures, these materials have direct band gaps in the visible-near IR range and provide large carrier mobility^5^, flexibility^6^, transparency, and fast lithium diffusion^7^. These properties make them suitable for digital circuits^8^, logic transistors^9^, light-emitting diodes (LEDs)^10^, photovoltaic devices^11^, chemical sensors^12^, and Li-ion battery anodes^13^ etc. These atomically thin TMDs are large-gap higher-order topological crystalline insulators, which are protected by $\hbox {C}_{3}$ rotational symmetry^14^. One of the critical aspect of TMDs is that they can be deposited onto flexible substrates and survive the stress and strain compliance of flexible supports^15^. These fascinating properties of TMDs originate from the quantum confinement and surface effects that arise during the transition of an indirect bandgap to a direct bandgap semiconductor when bulk materials are scaled down to their monolayers. The TMD monolayers are dynamically and thermodynamically stable compared to other 2D materials. Therefore, they were widely synthesized by various experimental methods, including chemical vapor deposition (CVD)^16^, physical vapor deposition (PVD)^17^, mechanical exploitation (ME)^18^, and hydrothermal synthesis^19^ etc. In addition to this, 2D Janus materials (such as 2D Janus TMDs) are also dragging tremendous attention due to their unique structural, physical, and chemical properties^20^, which differ from conventional 2D materials. Recent studies also demonstrate the successful synthesis of 2D Janus MoSSe and WSSe monolayers by modified CVD technique^21^. These materials offer a novel property due to their broken mirror symmetry with distinct atomic types on their upper and lower side. The general formula of artificial 2D Janus TMDs is MXY, where M is a transition metal atom, which is sandwiched between two different chalcogen atoms X, and Y. As a result of the different atomic radii and electronegativities of the X and Y elements, the charge distributions between M-X and M-Y layers are not uniform. The mirror asymmetry geometry offers a variety of new features including the intrinsic electric dipole moment, piezoelectricity, Rashba spin splitting, and unique excitonic behavior^22–24^. Both experimental and theoretical studies showed that MoSeTe and WSeTe monolayers display remarkable absorption capabilities in the infrared, visible, and ultraviolet regions^25^. There is a potential for photovoltaic and optoelectronic applications with these Janus monolayers. Moreover, the tunable Rashba spin splitting was observed in the WSeTe monolayer by using density functional theory (DFT) and the potential applications for spin-based devices^26^. Therefore, it is reasonable to consider the 2D TMDs and XSeTe (X = Mo, W) as ideal materials of heterostructures for applications in electronic and optoelectronic devices.

In order to optimize the performance of 2D materials, different strategies are available, including doping^27^, introduction of defects, strain/electric field engineering^28–30^, and heterojunctions. Interestingly, the heterostructures have great potential for future flexible devices and their applications^31,32^. The 2D van der Waals Heterstructures (vdWHs) are smart artificial materials composed of two similar or different types of monolayers. These heterostructures play an important role in the current semiconductor industry as they combine the advantages of each monolayer and also introduce new exciting properties due to the weak interlayer coupling. Some TMD heterostructures are also successfully synthesized in experiments such as $\hbox {MoS}_2$/$\hbox {WS}_2$^33^, $\hbox {MoSe}_2$/$\hbox {WSe}_2$^34^, and $\hbox {MoS}_2$/$\hbox {MoSe}_2$^35^ that also show ferroelectric and piezoelectric properties^36^. Furthermore, the combination of Janus monolayers with other 2D materials gives birth to versatile heterostructures with magnificent properties such as enhanced out-of-plane piezoelectric response, high carrier mobility, excellent optical, tunable electrical contact properties, and also high absorption coefficient. Moreover, some Janus heterostructures have been reported, such as MoSSe/WSSe^37^, MoSSe/$\hbox {WSe}_2$, graphene/MoSSe, and XSSe/Mg(OH)$_2$ (X = Mo, W) with excellent photosensitivity, rich electronic^38,39^ and thermal properties^40^. A rotation/twist angle ($\theta$) has been introduced between the monolayers as a new degree of freedom to enhance the performance of the heterostructure. This small interlayer twist can induce unconventional superconductivity, orbital ferromagnetism, quantum anomalous Hall effect, topological phases, and more interesting properties^41,42^. For example a distinguishable twist-induced plateau-like magnetoresistance ($\sim$ 0.05%) has been observed in 2D magnetic $\hbox {Fe}_{3}$ $\hbox {GeTe}_{2}$ homojunction at $\theta$ = 87$^\circ$^43^. The twist angle determines the new periodicity formed between the individual monolayers, while the interlayer coupling determines the magnitude of hybridization, charge redistribution, and lattice reconstruction. It is also possible to fabricate the required rotation angle in an experiment by transfer method and atomic force microscope (AFM) tip manipulation techniques.

Here, we created all possible combination of distinct inhomogeneous heterostructures using one layer of TMD ($\hbox {MoS}_{2}$, $\hbox {WS}_{2}$, $\hbox {MoSe}_{2}$, and $\hbox {WSe}_{2}$) stacked on the top of Janus monolayers (MoSeTe or WSeTe), inspired by the magical interplay of the twist angles in the heterostructure and their successful synthesization. We introduce different combination of Janus and TMDs heterostructure of: $\hbox {MoS}_2$/MoSeTe, $\hbox {MoS}_2$/WSeTe, $\hbox {WS}_2$/MoSeTe, $\hbox {WS}_2$/WSeTe, $\hbox {MoSe}_2$/MoSeTe, $\hbox {MoSe}_2$/WSeTe, $\hbox {WSe}_2$/MoSeTe, $\hbox {WSe}_2$/WSeTe, and MoSeTe/WSeTe; and systematically investigated their electrical and optical response as a function of twist angle. The TMD monolayers form a well-matched interface with the Janus layer due to their structural similarity. Further, their electronic band gaps and the band edge positions are suitable for visible light photocatalysis. Direct bandgaps coupled with Type-II alignments make these materials ideal for high-efficiency photovoltaic materials. The high carrier mobility and excellent absorption coefficient make these heterostructures more useful for future nanoelectronic and optoelectronic devices.

## Methodology

First-principles based density functional theory (DFT) calculations were performed using the Quantum ATK^44^, using the projector augmented wave (PAW) method. The plane-wave cut-off energy is set to 790 eV and the Monkhorst-Pack^45^ mesh of k-points with 9 $\times$ 9 $\times$ 1 and 11 $\times$ 11 $\times$ 1 points are used to sample the Brillouin zone for monolayers and heterostructures, respectively. A 15 Å vacuum layer along the z-direction is employed to avoid interactions between the neighboring layers. The lattice constant and atomic positions are relaxed until the force on each atom is less than 0.01 eV/Å. The convergence criterion of the total energy is set at 10$^{-5}$ eV/atom. The phonon band structure was calculated with the finite displacement method by using the PHONOPY code^46^, where a 3 $\times$ 3 $\times$ 1 supercell was used. The Kubo–Greenwood formula^47^ was used to calculate the susceptibility tensor for estimating the optical spectrum as given in Eq. (1).

1 $$\begin{aligned} \small {\chi _{ij}(\omega ) = \frac{e^2}{\hbar m_e^2 V}\sum \limits _{nm}\frac{f_{mk} - f_{nk}}{\omega ^2_{nm}(k)\bigg [\omega _{nm}(k) - \omega - \frac{i\delta }{\hbar }\bigg ]}p^i_{nm}(k)p^j_{mn}(k)}. \end{aligned}$$

Here, $p^i_{nm}$ and $p^j_{mn}$ belong to *i*th and *j*th component of the dipole matrix element between state *n* and *m* respectively, V is the volume of the material, $\delta$ is broadening and $f_{nk}$ indicates Fermi function evaluated at the band energy $E_n(k)$.

## Results and discussion

In this present study, we have considered two Janus (MoSeTe and WSeTe) monolayers and four TMD monolayers ($\hbox {MoS}_2$, $\hbox {MoSe}_2$, $\hbox {WS}_2$ and $\hbox {WSe}_2$). We have systematically investigated the stability, electrical, and optical properties of Janus MoSeTe and WSeTe monolayers, as well as vertically stacked heterostructures, made up of Janus monolayers and TMDs. The weak van der Waals forces between the monolayers do not cause substantial atomic-scale alterations and usually maintain the original features of the monolayer in the heterostructure. Hence, the combination of two different monolayers into a heterostructure can offer some unique and fascinating properties as compared to its monolayer.

**Figure 1:**
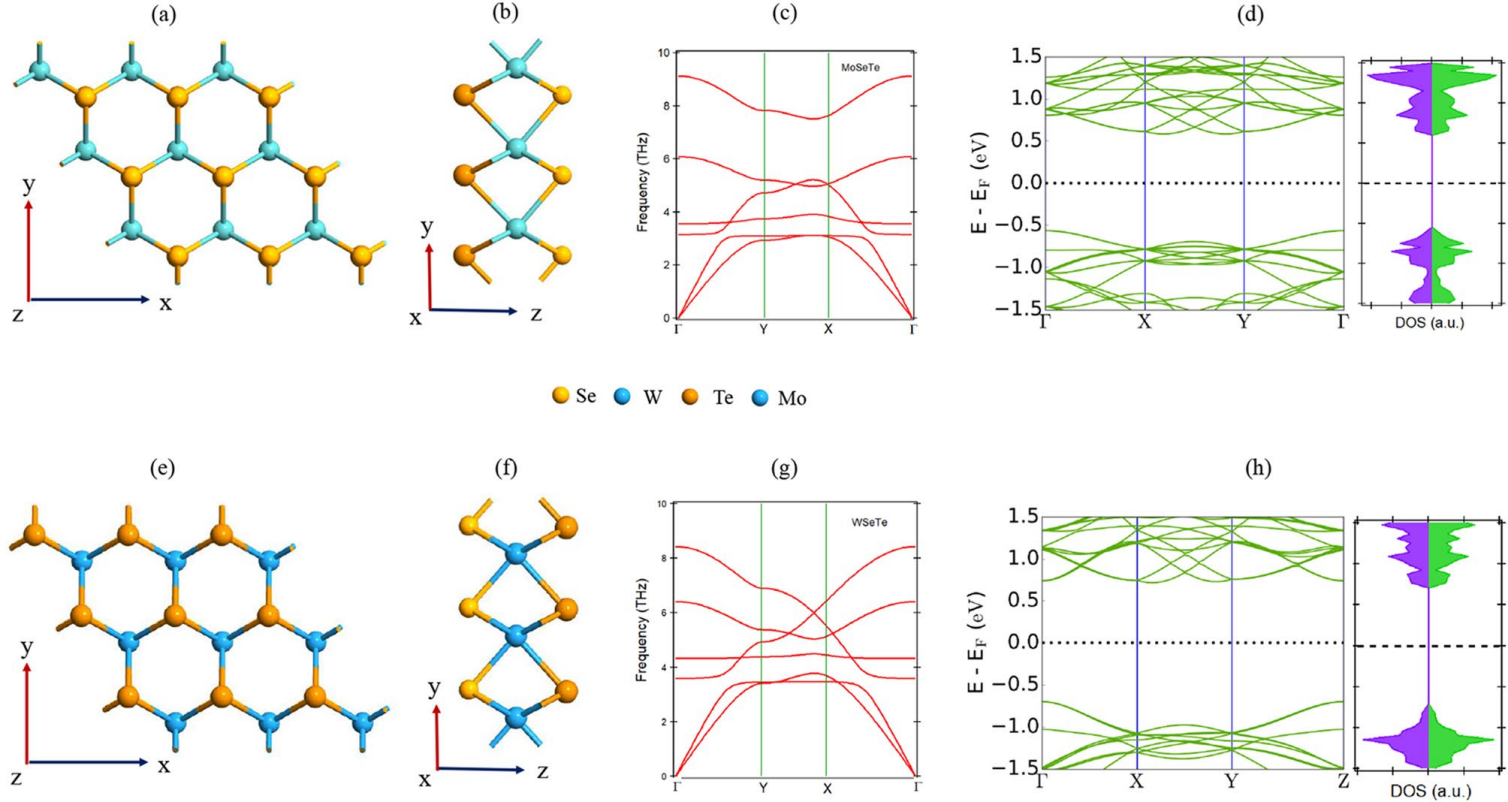
(**a,b,e,f**) Represent the top, and side view, (**c,g**) indicate the phonon dispersion curve, and (**d,h**) represents the bandstructure and density of states (DOS) of Janus MoSeTe and WSeTe monolayer respectively. The green and purple color represent the spin-$\uparrow$ and spin-$\downarrow$ states, respectively. The black dotted line stands for the Fermi level.

### Structural and electronic properties of the monolayers

The top and side views of the optimized Janus MoSeTe and WSeTe monolayers are shown in Fig. 1a,b,e,f. The structure of Janus monolayers (XSeTe, X = Mo, and W) is constructed in three planes, where the X atom is sandwiched between the layer composed of Se and Te layers. The bond length between the Mo–Se, Mo–Te, W–Se, and W–Te are different due to the difference in atomic number between the atoms. The TMDs monolayers ($\hbox {XY}_2$, X = Mo, W, and Y = S, Se) also consist of three layers, where the X layer is stacked between two similar Y layers (as shown in Fig. S1 in the supplementary information (SI)). The computed lattice parameters for all the TMDs and Janus monolayers are presented in Table S1 of the SI. The phonon dispersion of Janus MoSeTe and WSeTe monolayer is plotted in Fig. 1c,g, respectively, which shows no imaginary phonon modes, suggesting that monolayers are dynamically stable. The four TMD monolayers are very well-known 2D materials, with earlier research indicating that they are dynamically stable^48–50^. The optical phonon vibration modes of the Janus monolayers at the $\Gamma$ point can be defined as:

2 $$\begin{aligned} \Gamma _{optical} = A_2^{''}(IR) + A_1^{'}(R) + E^{'}(IR + R) + E^{''}(R). \end{aligned}$$

Here, the IR and R represent infrared and Raman active mode, respectively. The optical phonon frequencies of Janus MoSeTe are 11.46 THz ($A_2^{''}$), 8.18 THz ($A_1^{'}$), 5.42 THz ($E^{'}$) and 4.45 THz ($E^{''}$). Similarly the phonon frequencies for Janus WSeTe are 8.42 THz ($A_2^{''}$), 6.41 THz ($A_1^{'}$), 4.32 THz ($E^{'}$) and 3.58 THz ($E^{''}$). Further, we have computed the electronic properties of all the monolayers. The bandstructure and density of states of the Janus MoSeTe and WSeTe monolayers are presented in Fig. 1d,h, respectively. Our calculated result demonstrates that the Janus monolayers are indirect band gap semiconductor, whereas the TMDs are direct bandgap semiconductors. We have also calculated the band structure and absorption coefficient of monolayer TMDs through HSE method (Figs. S2, S15 in SI), which shows the semiconducting behaviour with high band gap values in comparison to PBE calculation. The bandgap value and its nature are tabulated in Table S1 of the SI. As shown in Fig. S3a in the SI, the projected density of states (PDOS) of MoSeTe shows that $d_{xy}$ (Mo) is the main contributor to the valence band (VB), with small contributions from the $p_x$ (Te) states, whereas the conduction band (CB) originates from the $p_z$ (Te) and $d_{yz}$ (Mo) states. In the case of WSeTe monolayer, the valence band mainly originates from the $d_{xy}$ (W) and $p_y$ (Te) states, but the conduction band is mostly contributed by the $d_{x^2-y^2}$ (Te) with a negligible contribution of $p_y$ (Te).

### Heterostructures and their properties

We have proposed nine different types of vertically stacked heterostructure (TMD/Janus: 8 and Janus/Janus: (1) since the TMDs and Janus monolayers are well-studied material for electronic and optoelectronic applications. In addition, the structural similarity between the TMDs and Janus monolayers allows the formation of better quality interface. All the nine vdWHs are: $\hbox {MoS}_2$/MoSeTe, $\hbox {MoS}_2$/WSeTe, $\hbox {WS}_2$/MoSeTe, $\hbox {WS}_2$/WSeTe, $\hbox {MoSe}_2$/MoSeTe, $\hbox {MoSe}_2$/WSeTe, $\hbox {WSe}_2$/MoSeTe, $\hbox {WSe}_2$/WSeTe, and MoSeTe/WSeTe. In addition to this, the relative twist angle between the monolayers also gives rise to lattice reconstruction and enhances the performance of the vdWHs due to the complex interlayer coupling. We considered all the possible twist angles between the monolayers in the range of 0–60$^{\circ }$. The twist angles were chosen based on the number of atoms present in the supercell of the heterostructure and the minimum lattice mismatch between the monolayers. All nine heterostructures fall into two categories, allowing four rotation angles for $\hbox {XSe}_2$/XSeTe (X = Mo, W) and MoSeTe/WSeTe and three rotation angles for $\hbox {XS}_2$/XSeTe (X = Mo, W) heterostructure, by satisfying the above criteria. Figure S4a in the SI shows the top and side views of the Janus MoSeTe/WSeTe heterostructure at $\theta$ = 0$^{\circ }$, whereas Fig. S5a–d in the SI represents additional schematic representations of the heterostructure for different rotation angles. We have also calculated the interlayer distance for each rotation angle by considering the minimum energy criteria. An example of estimation of interlayer distance for Janus $\hbox {WSe}_2$/WSeTe vdWH is shown in Fig. S6 in the SI. The estimated number of atoms present in the supercell, lattice mismatch, and interlayer distance for Janus $\hbox {XS}_2$/XSeTe, $\hbox {XSe}_2$/XSeTe, and MoSeTe/WSeTe vdWHs are presented in the SI of Tables S2–S4 respectively.

#### Stability

**Figure 2:**
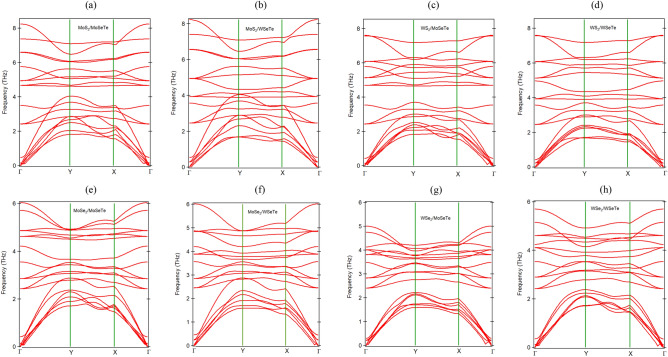
The phonon dispersion of the Janus vdWHs at $\theta$ = 0$^{\circ }$: (**a**) $\hbox {MoS}_2$/MoSeTe, (**b**) $\hbox {MoS}_2$/WSeTe, (**c**) $\hbox {WS}_2$/MoSeTe, (**d**) $\hbox {WS}_2$/WSeTe, (**e**) $\hbox {MoSe}_2$/MoSeTe, (**f**) $\hbox {MoSe}_2$/WSeTe, (**g**) $\hbox {WSe}_2$/MoSeTe, and (**h**) $\hbox {WSe}_2$/WSeTe.

The dynamical stability of 2D vdWHs plays a very vital role in its practical application in nanoelectronic and optoelectronic devices. We have calculated phonon bandstructure along the high symmetric points ($\Gamma$–Y–X–$\Gamma$) for all the Janus vdWHs at $\theta$ = $0^{\circ }$. The phonon band structures of Janus $\hbox {XS}_2$/XSeTe and $\hbox {XSe}_2$/XSeTe vdWHs are presented in Fig. 2a–h and Janus MoSeTe/WSeTe vdWH is shown in Fig. S4b in the SI. The absence of imaginary vibration frequencies confirms the dynamical stability of the heterostructure. Interestingly, the soft acoustic mode of Janus MoSeTe and WSeTe monolayer has a frequency of 3 THz and 3.3 THz at the Y-point, respectively. This frequency value becomes softer when the Janus monolayers from a heterostructure with TMDs monolayer.

**Figure 3:**
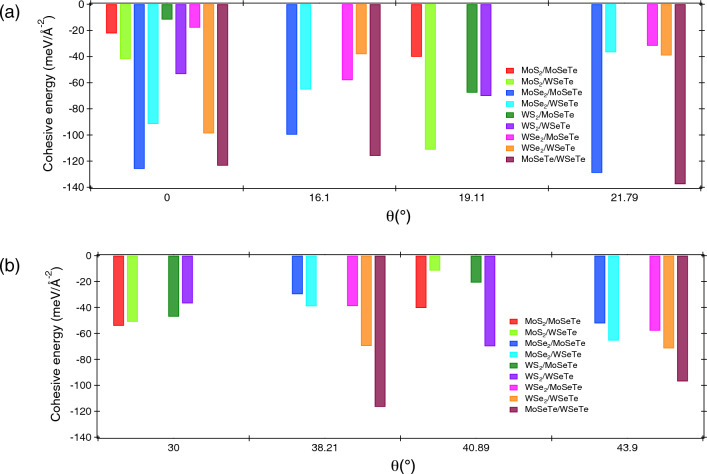
The variation of cohesive energy of Janus vdWHs as a function of rotation angles.

To address the thermodynamic stabilities of the Janus heterostructure, the interface cohesive energies were calculated using Eq. (3).

3 $$\begin{aligned} E_{CE} = E_{X/Y} - E_X - E_Y. \end{aligned}$$

Here, $\hbox {E}_{CE}$ and $E_{X/Y}$ represent the cohesive energy and total energy of the vdWHs, respectively. The $E_X$ and $E_Y$ show the energy of the constituent monolayers. The variation of cohesive energy for all the Janus vdWHs at different rotation angles is presented in Fig. 3. The low value of cohesive energies indicates that the Janus and TMD monolayers can form a stable interface. The Janus vdWHs are quite favorable because all TMDs and Janus monolayers have comparable structural geometry. The minimum cohesive energy obtained for Janus MoSeTe/WSeTe vdWHs is − 137.56 meV/Å$^{-2}$ at $\theta$ = 21.79$^\circ$. It is worth mentioning that the cohesive energy is minimum, at which the lattice mismatch is also minimum, allowing each monolayer to make a better and strongly coupled interface. Overall, the cohesive energy varies between 11 and 138 meV/Å$^{-2}$ for all the Janus vdWHs. This value is also well comparable with other similar type of 2D heterostructures such as XSSe/Mg(OH)$_2$ (X = Mo, W)^51^, Graphene/$\hbox {MoS}_2$^52^, and $\hbox {WTe}_2$/Graphene^53^. To check the mechanical stability, we computed the elastic constants of Janus vdWHs using the Born–Huang criteria^54^. The estimated elastic constant is in N/m unit, which explains the elastic potential of Janus vdWHs. The estimated magnitude of elastic constant of MoSeTe/WSeTe at $\theta$ = 0$^{\circ }$ is: $\hbox {C}_{11}$ = 42.90 N/m, $\hbox {C}_{12}$ = 9.04 N/m, $\hbox {C}_{13}$ = 0.14 N/m, $\hbox {C}_{14}$ = − 0.05 N/m, $\hbox {C}_{15}$ = − 0.05 N/m, $\hbox {C}_{16}$ = − 0.23 N/m, $\hbox {C}_{22}$ = 43.18 N/m, $\hbox {C}_{23}$ = 0.08 N/m, $\hbox {C}_{24}$ = 0.15 N/m, $\hbox {C}_{25}$ = 0.10 N/m, $\hbox {C}_{26}$ = − 0.13 N/m, $\hbox {C}_{33}$ = 0.37 N/m, $\hbox {C}_{34}$ = 0.08 N/m, $\hbox {C}_{35}$ = − 0.18 N/m, $\hbox {C}_{36}$ = 0.12 N/m, $\hbox {C}_{44}$ = 0.34 N/m, $\hbox {C}_{45}$ = 0.15 N/m, $\hbox {C}_{46}$ = 0.02 N/m, $\hbox {C}_{55}$ = 0.39 N/m, $\hbox {C}_{56}$ = 0.02 N/m, $\hbox {C}_{66}$ = 16.88 N/m. The variation of Young’s modulus and Poission’s ratio for Janus vdW Heterostructures at $\theta$ = 0$^{\circ }$ is illustrated in Figs. S13 and S14 in the SI. It is observed that Young’s modulus is higher along the x-direction than in the y and z directions. There was a maximum value of 137.71 N/m for $\hbox {MoS}_{2}$/WSeTe, which is lower than $\hbox {MoSi}_{2}$ $\hbox {As}_{2}$ monolayer^55^, but comparable to other 2D materials, such as $\hbox {Ti}_{2}$C (137 N/m)^56^, $\hbox {B}_{2}$S (137 N/m to 143 N/m)^57^, $\hbox {TiC}_{2}$ (131.17 N/m)^58^ and higher than the Phosphorene (92.4 N/m)^59^, silicene (62 N/m)^57^. The ZX and ZY components of Poission’s ratio has a higher value along the in-plane directions, where the highest value has been estimated to be 0.2949, which is approximately the same as that of monolayer $\hbox {MoSi}_{2}$ $\hbox {As}_{4}$^55^.

#### Electronic properties

**Figure 4:**
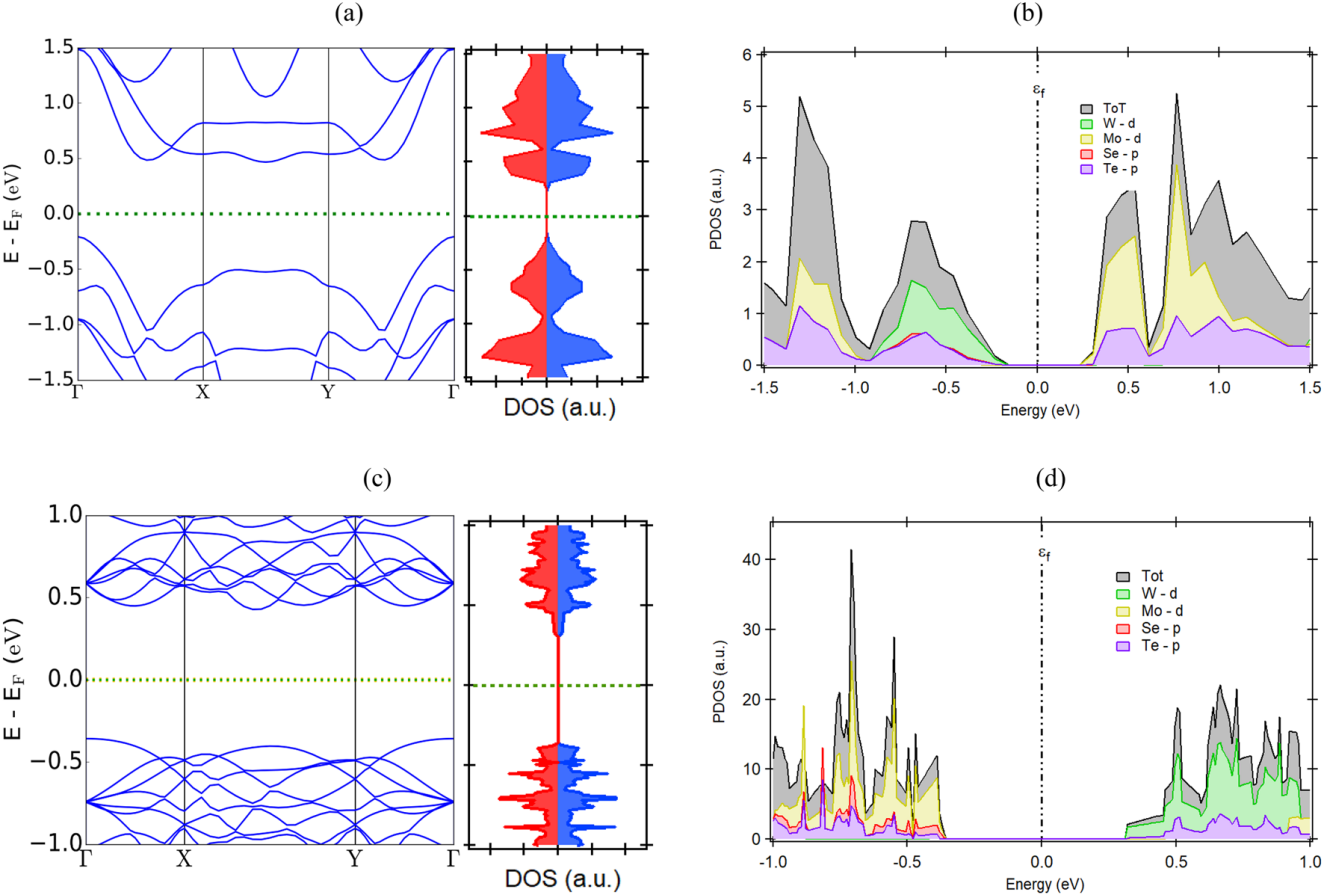
The bandstructure and DOS of Janus MoSeTe/WSeTe vdWHs at: (**a,b**) $\theta$ = $0^{\circ }$ and (**c,d**) $\theta$ = $21.79^\circ$.

**Figure 5:**
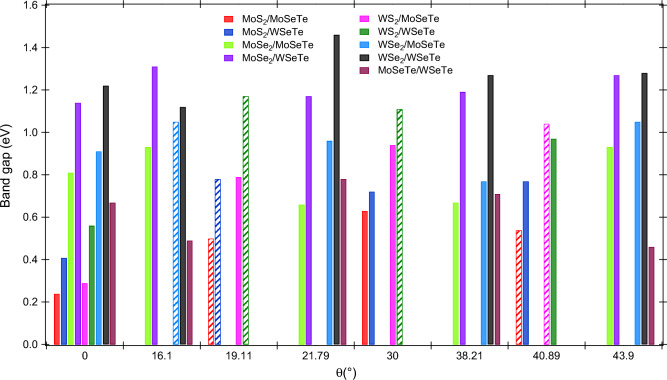
The variation of bang gap at different rotation angles for the Janus vdWHs. The solid and patterned bar represents the indirect and direct bandgap, respectively.

The energy band structure of the Janus vdWHs is critically important for determining the performance of the material for different technological applications. The band structures of Janus MoSeTe/WSeTe vdWH at $\theta$ = 0$^{\circ }$ and 21.79$^{\circ }$ are calculated and presented in Fig. 4a,c respectively. The Janus MoSeTe/WSeTe vdWH has the same indirect bandgap semiconducting property as monolayers but with a reduced value. The calculated band gaps for this vdWHs are 0.67, 0.49, 0.78, 0.71, and 0.46 eV at $\theta$ = $0^{\circ }$, $16.10^{\circ }$, $21.79^{\circ }$, $38.21^{\circ }$, and $43.90^{\circ }$ respectively. To characterize the behavior of electrons at the band edges, the projected density of states (PDOSs) has been calculated and plotted in Fig. 4b,d. The PDOS analysis reveals that at $\theta$ = $0^{\circ }$, the VBM is dominated by the $\hbox {d}_{x^2-y^2}$ orbital of W, and the CBM is contributed by the $\hbox {d}_{z^2}$ orbital Mo atom. In contrast, the reverse nature of PDOS was observed for the rest of the rotation angles, which means the VBM and CBM reside on d-orbitals of Mo and W, respectively. The origin of the CBM and VBM is due to the presence of two different Janus monolayers, implying the MoSeTe/WSeTe vdWHs have type-II band alignment with the staggered gaps, which is also supported by the projected band structure of the WSeTe/MoSeTe heterostructure ($\theta$ = $0^{\circ }$, as illustrated in Fig. S11a in the SI), in which orange band lines represent the contribution of the WSeTe layer and blue lines indicate the MoSeTe contribution. We can easily see that WSeTe creates valence band maxima, while MoSeTe creates conduction band minima. As shown in Fig. S11b,c in SI, the partial charge density above and below the Fermi level also suggested type-II band alignment. A dense cloud of electrons surround the W atom below the Fermi level, while a cloud of electrons surround the Mo atom above the Fermi level. Such materials are beneficial for photocatalytic water splitting and enable high-efficiency solar energy conversion. The variation of band gap for all the Janus vdWHs at different twisting angles are shown in Fig. 5. The bandgap values of Janus vdWHs lie between 0.2 and 1.5 eV. The energy bandstructure along the high symmetric paths for the two different classes of Janus vdWHs ($\hbox {MoS}_2$/MoSeTe and $\hbox {WSe}_2$/WSeTe) are depicted in Figs. S7 and S8 in the SI, respectively. Mostly, the CBM lies in between X and Y-points of the BZ for all twisting angles, but the VBM resides on X-point for $\theta$ = $0^{\circ }$ and at $\Gamma$-point for the rest of the twisting angles. The maximum bandgap obtained is 1.46 eV for Janus $\hbox {WSe}_2$/WSeTe vdWH at $\theta$ = 21.79$^{\circ }$. Similarly, we observed a minimum band gap of 0.24 eV for Janus $\hbox {MoS}_2$/MoSeTe vdWH at $\theta$ = 0$^{\circ }$. We also observed that the Janus $\hbox {XS}_2$/XSeTe vdWHS have a lower bandgap as compared to Janus $\hbox {XSe}_2$/XSeTe vdWHs. This may be due to the increased bond length and atomic number between X-S to X-Se. As shown in Fig. S9 in the SI, the band diagram of MoSeTe/WSeTe heterostructure at $\theta$ = $0^{\circ }$ includes vdW and dipole corrections. The band structure shows semiconducting behavior with an indirect bandgap of 0.67 eV, which is approx. equal to the PBE calculation. We have also compared the bandgap of Janus vdW heterostructures at $\theta$ = $0^{\circ }$ between the PBE calculation and vdW + dipole corrections, as shown in Fig. S10 in the SI. We have not found any significant difference in the electronic structure for all 9 Janus vdW heterostructures between PBE calculation and PBE + vdW + dipole correction. The PDOS analysis confirms that all the Janus vdWHs at different rotation angle have type-II band alignment. Surprisingly, $\hbox {MoS}_2$/MoSeTe ($\theta$ = 19.11$^{\circ }$, 40.89$^{\circ }$), $\hbox {WS}_2$/WSeTe ($\theta$ = 19.11$^{\circ }$, 30$^{\circ }$), $\hbox {MoS}_2$/WSeTe ($\theta$ = 19.11$^{\circ }$), $\hbox {WSe}_2$/MoSeTe ($\theta$ = 16.10$^{\circ }$), $\hbox {WS}_2$/MoSeTe ($\theta$ = 40.89$^{\circ }$) have direct bandgap with type-II band alignment, which is highly desirable to enhance the efficiency of photovoltaic solar cells. The bandstructure difference among the vdWHs mainly arises from the change in interlayer coupling induced by the twisting angle. The Janus vdWHs show an electronic phase cross-over from direct to indirect bandgap semiconductor since the hybridization of atomic orbitals changes significantly at the Fermi level due to the interlayer rotation. The hybridization extent determines the value of the band gap. The lower band gap of the Janus vdWHs can improve the optical absorption in visible light and near-ultraviolet regions. The main reason behind the change in bandgap for Janus vdWHs at different rotation angles can be described by calculating interlayer charge transfer.

**Figure 6:**
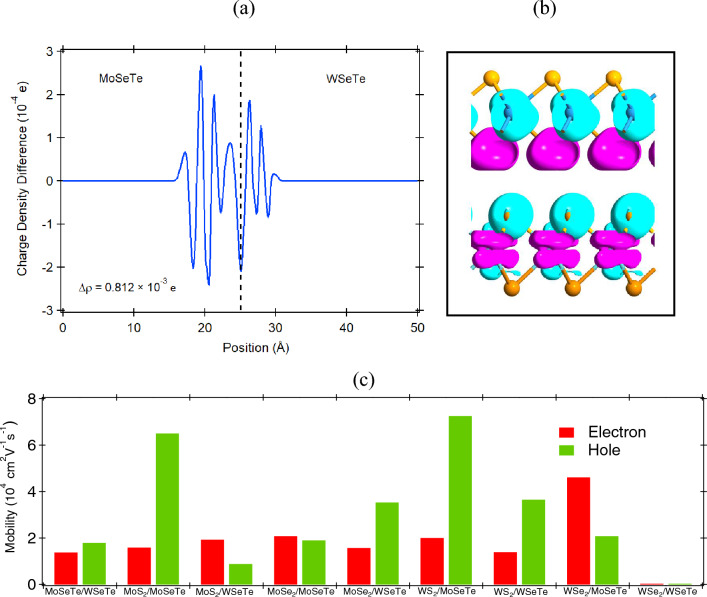
(**a**) Plane-integrated charge density difference and (**b**) three-dimensional (3D) charge density difference with the isosurface value of 4.9 $\times$ 10^−5^ eÅ$^{-3}$ of Janus MoSeTe/WSeTe vdWH at $\theta$ = 0$^{\circ }$ . The dash-dot lines represent the interface. The cyan and pink color represent the charge depletion and accumulation respectively. (**c**) The variation of mobility of the Janus vdWHs at $\theta$ = 0$^{\circ }$.

To understand the charge transfer in the Janus vdWHs, we plot the charge density difference, as shown in Fig. 6a. The planar-averaged charge density difference is calculated using the Eq. (4).

4 $$\begin{aligned} \Delta \rho (z) = \int \rho _{_{H}}(x, y, z)dxdy - \int \rho _{_{MoSeTe}}(x, y, z)dxdy - \int \rho _{_{WSeTe}}(x, y, z)dxdy. \end{aligned}$$

Here, $\rho _{_{H}}(x, y, z)dxdy$, $\rho _{_{MoSeTe}}(x, y, z)dxdy$, and $\rho _{_{WSeTe}}(x, y, z)dxdy$ are the charge density at the (x,y,z) point for the Janus MoSeTe/WSeTe vdWHs, MoSeTe, and WSeTe monolayers respectively. Based on the Bader charge analysis, the calculated value of the charge transfer for Janus MoSeTe/WSeTe heterostructure at $\theta$ = $0^{\circ }$ through the interface region is estimated at $\Delta \rho$ = 0.812 $\times$ 10$^{-3}$ e (as shown in Fig. 6a). The positive values denote charge accumulation, and the negative values denote charge depletion. The results show that a charge rearrangement occurs, and electrons accumulate at the interface. We also calculated the Barder charge for all the heterostructures at different twist angles and found that the value is positive for all the twist angles. To get a clear picture of charge accumulation at the interface, we have plotted isosurface plot, as shown in Fig. 6b. The cyan and pink color represent the charge depletion and accumulation, respectively. As can be seen, the charge accumulation mostly occurs near the interface region of MoSeTe and WSeTe monolayer. However, the charge redistribution strength gets decreased as we further move away from the interface region. This is due to the weak van der Waals interaction between the Janus monolayers. The accumulated charge forms an interlayer electric field, which ultimately affects the charge transfer between the monolayers. The most significant difference between the interlayer electronic interaction in Janus vdWHs is that there is discernible interlayer charge transfer due to the presence of different X and chalcogen atoms in monolayers. The interlayer charge transfer will induce an electrostatic interlayer interaction, which will give rise to an energy level shift in opposite directions for the two constituent monolayers. Furthermore, the twisting angle, interlayer distance, lattice mismatch, and geometry of the Janus vdWHs determine the amount of charge transfer between the monolayers. These Janus vdWHs have direct and indirect bandgap with type-II band alignment to facilitate the separation of electrons and holes, which is useful for optoelectronic and photovoltaic devices^60^.

The higher carrier mobility is another important indicator for determining the performance of the heterostructure in photovoltaic and photocatalytic applications. We have quantitatively estimated the carrier mobility for the Janus vdWHs in the x-direction by using the acoustic phonon limited method by using Eq. (5).

5 $$\begin{aligned} \mu _{2D} = \frac{e\hbar ^3C_{2D}}{K_BTm^*m_dE_l^2}. \end{aligned}$$

Here, T is the temperature, $m^*$ is the effective mass in the transport direction, and $m_d$ = $\sqrt{m_{x}m_{y}}$ is the average effective mass. $C_{2D}$([$\frac{\partial ^2E}{\partial ^2\delta }$]/$S_0$) is the elastic modulus. E is the total energy after applying strain ($\delta$ = $\Delta$l/$l_0$) and $S_0$ is the area at equilibrium. In addition $E_l$ ($\Delta$E/$\delta$) is the diffusion constant, where $\Delta$E is the energy shift of the band edge of CBM or VBM with respect to the vacuum level. The estimated electron mobility of the Janus MoSeTe and WSeTe monolayers are 1.07 $\times$ 10^4^ and 2.33 $\times$ 10^4^ $\hbox {cm}^2$/Vs and for holes are 3.11 $\times$ 10^4^ and 7.79 $\times$ 10^4^ $\hbox {cm}^2$/Vs respectively. The hole mobilities of Janus monolayers are higher than their electron mobilities due to the relatively smaller deformation potentials. Earlier studies revealed that the conventional TMD monolayers have low carrier mobility^61,62^. The electron mobilities of the Janus vdWHs can also be enhanced when the Janus monolayer is stacked into vertical heterostructures with TMDs monolayer. High electron mobility ($\sim$ 10^4^ $\hbox {cm}^2\, \hbox {V}^{-1}\, \hbox {s}^{-1}$) have been observed for $\hbox {MoS}_2$/WSeTe, $\hbox {MoSe}_2$/MoSeTe, and $\hbox {WSe}_2$/WSeTe vdWHs, as well as high hole mobility ($\sim$ 10^4^  $\hbox {cm}^2\, \hbox {V}^{-1}\, \hbox {s}^{-1}$) for MoSeTe/WSeTe, $\hbox {MoS}_2$/MoSeTe, $\hbox {MoSe}_2$/WSeTe, and $\hbox {WS}_2$/MoSeTe vdWHs. The variation of electron and hole mobilities for Janus vdWHs at $\theta$ = 0$^{\circ }$ is depicted in Fig. 6c. Moreover, high and low electron mobilities reach 4.61 $\times$ 10^4^ $\hbox {cm}^2$/Vs for Janus $\hbox {WSe}_2$/MoSeTe and $\hbox {WS}_2$/WSeTe vdWH at $\theta$ = 0$^{\circ }$ respectively. These calculated carrier mobilities are quite high as compared to conventional 2D vdWHs. The high carrier mobility in vdWHs is attributed to the low effective mass (Fig. S12 in SI) and more dispersed band at the Fermi level, which would induce fast migration of the carriers and suppress the recombination of photo generated electron and hole pairs.

**Figure 7:**
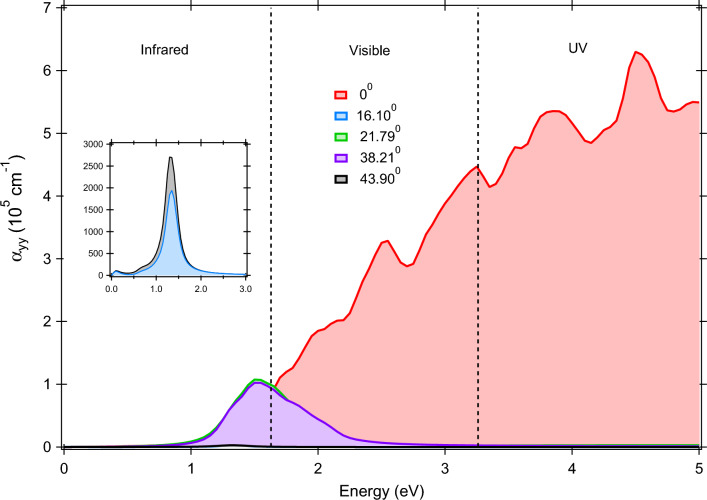
The optical absorption spectra for Janus MoSeTe/WSeTe vdWHs at different rotation angles.

#### Optical properties

The optical properties of the vertically stacked Janus vdWHs at different twisting angles are studied by computing the complex dielectric function as given below,

6 $$\begin{aligned} \varepsilon (\omega ) = \varepsilon _1(\omega ) + i\varepsilon _2(\omega ). \end{aligned}$$

Here, $\varepsilon _1$($\omega$) and $\varepsilon _2$($\omega$) represents the real and imaginary part of dielectric constant respectively. The $\varepsilon _2$($\omega$) can be calculated by summing all the empty states in the Brillouin zone, while the $\varepsilon _1$($\omega$) of the dielectric function can be obtained by using the Kramers–Kronig relation^63^. We have calculated the optical properties in the energy window of 0–5 eV for all the Janus vdWHs. The dielectric function is calculated along xx/yy (E $\perp$ z) and zz (E $\parallel$ z) directions. All the Janus vdWHs exhibit strong anisotropic nature due to their crystal symmetry, but we have considered only the maximum contribution, which is along yy-directions for the majority of the case.

The real static part of the dielectric function is the direct measure of refractive index (n). The higher the value of n, the better will be the probability of absorbing light. The refractive index is 1.27, 1.51, 2.84, 2.68, 2.63, and 2.38 for MoSeTe, WSeTe, $\hbox {MoS}_2$, $\hbox {MoSe}_2$, $\hbox {WS}_2$, and $\hbox {WSe}_2$ monolayers respectively. Table 1 lists the estimated refractive index for nine Janus vdWHs at various rotation angles. The value of *n* behaves differently for different heterostructures as well as rotation angles. We have also observed that the maximum enhancement in *n* is at $\theta$ = 0$^{\circ }$ for all the Janus vdWHs. This enrichment is due to the perfect interlayer coupling between the TMDs and Janus monolayers at that rotation angle. In case of Janus MoSeTe/WSeTe vdWHs, the *n* increases up to a value of 3.87 at $\theta$ = 0$^{\circ }$. The rotation angles give an extra freedom to use the same material for different applications with a low and high value of the refractive index. The low value of refractive index materials has a great potential in many photonic device applications due to their high reflective behavior, whereas the high value of refractive index materials are useful in storing energies such as solar cell. The imaginary part of the dielectric function reflects the transitions from occupied to unoccupied bands. The optical absorption coefficients of the heterostructures are obtained using the Eq. (7):

7 $$\begin{aligned} \alpha (\omega ) = \sqrt{2}\omega \Bigl [\sqrt{\varepsilon _1^2(\omega ) + \varepsilon _2^2(\omega )} - \varepsilon _1(\omega )\Bigr ]^ {\frac{1}{2}}. \end{aligned}$$

Here, $\omega$ and $\alpha$ are utilized to express angular frequency and absorption coefficient.

**Table 1:** The variation of refractive index (n) at different rotation angle ($\theta$) of Janus vdWHs.

$\theta$($^{\circ }$)	Refractive index								
	$\hbox {MoS}_2$/MoSeTe	$\hbox {MoS}_2$/WSeTe	$\hbox {MoSe}_2$/MoSeTe	$\hbox {MoSe}_2$/WSeTe	$\hbox {WS}_2$/MoSeTe	$\hbox {WS}_2$/WSeTe	$\hbox {WSe}_2$/MoSeTe	$\hbox {WSe}_2$/WSeTe	MoSeTe/WSeTe
0	5.89	5.53	5.62	5.53	6.00	5.25	5.28	1.75	3.87
16.10	–	–	1.34	1.62	–	–	1.75	1.65	1.10
19.11	1.7	1.84	–	–	2.05	2.28	–	–	–
21.79	–	–	2.0	2.02	–	–	2.26	2.12	1.15
30	1.83	2.20	–	–	2.09	3.24	–	–	–
38.21	–	–	2.06	2.02	–	–	2.25	2.12	1.02
40.89	1.57	1.81	–	–	2.81	2.28	–	–	–
43.90	–	–	1.26	1.62	–	–	1.75	1.66	1.06

The absorption coefficient for Janus MoSeTe/WSeTe heterostructure is plotted in Fig. 7. The starting point of the absorption edge represents the optical bandgap which means the minimum amount of energy required for the transition of electron from VBM to CBM. For Janus $\hbox {XS}_2$/XSeTe vdWHs, the order of absorption coefficient with respect to twist angle is $\theta$ = 0$^{\circ }$ > 30$^{\circ }$ > 40.89$^{\circ }$ > 19.11$^{\circ }$, whereas the order is 0$^{\circ }$ > 21.79$^{\circ }$ > 38.21$^{\circ }$ > 43.90$^{\circ }$ > 16.10$^{\circ }$ for Janus $\hbox {XSe}_2$/XSeTe (MoSeTe/WSeTe) vdWHs. Thus, we can conclude that the maximum contribution of absorption coefficient is at $\theta$ = 0$^{\circ }$ for all the Janus vdWHs. Figure S16 in the SI represents the variation of absorption coefficients at $\theta$ = 0$^{\circ }$ for a photon energy of 3 eV. The maximum absorption coefficient obtained is 4.34 $\times$ 10$^5$ $\hbox {cm}^{-1}$ for Janus $\hbox {WSe}_2$/MoSeTe VdWH at $\theta$ = 0$^{\circ }$, enabling the Janus vdWH to be a promising solar light absorber material in the visible region. This value of absorption coefficient is also quite high as compared to other 2D heterostructures. This blue shifting nature of the abortion spectrum at $\theta$ = $0^{\circ }$ is due to the weak interlayer coupling and the formation of a suitable interface between the monolayers. Other twisting angles such as 16.10$^{\circ }$, 19.11$^{\circ }$, 21.79$^{\circ }$, 30$^{\circ }$, 38.21$^{\circ }$, 40.89$^{\circ }$, and 43.90$^{\circ }$ are active in between 1 and 3 eV and thereafter become completely transparent. The absorption peaks arise due to the electronic transition from the occupied states to the empty states. For example, The transition from $\hbox {d}_{x^2-y^2}$ (W) to $\hbox {d}_{z^2}$ (Mo) in MoSeTe/WSeTe ($\theta$ = 0$^{\circ }$) vdWH resulted in a prominent peak at a photon energy of 2.6 eV. The value of the absorption coefficient also well matches the refractive index. As we can see from Table 1, with the twist between the monolayers, the refractive index decreases, which results into the low value of the absorption coefficient. The optical properties of the Janus vdWHs completely depend on the hybridization of orbitals at the Fermi level due to the interlayer coupling. Furthermore, the Janus $\hbox {MoS}_2$/MoSeTe ($\theta$ = 19.11$^{\circ }$, 40.89$^{\circ }$), $\hbox {WS}_2$/WSeTe ($\theta$ = 19.11$^{\circ }$, 30$^{\circ }$), $\hbox {MoS}_2$/WSeTe ($\theta$ = 19.11$^{\circ }$), $\hbox {WSe}_2$/MoSeTe ($\theta$ = 16.10$^{\circ }$), and $\hbox {WS}_2$/MoSeTe ($\theta$ = 40.89$^{\circ }$) vdWHs possess high absorption coefficient ($\sim$ 10$^5$) in the infrared region with direct bandgap, which is an essential requirement for infrared sensors. Therefore, the relative twist between the monolayers provides a new route to manipulate the properties of a heterostructure.

## Conclusion

In summery, we have systematically studied the stability, electronic, and optical properties of nine different Janus vdWHs ($\hbox {MoS}_2$/MoSeTe, $\hbox {MoS}_2$/WSeTe, $\hbox {WS}_2$/MoSeTe, $\hbox {WS}_2$/WSeTe, $\hbox {MoSe}_2$/MoSeTe, $\hbox {MoSe}_2$/WSeTe, $\hbox {WSe}_2$/MoSeTe, $\hbox {WSe}_2$/WSeTe, and MoSeTe/WSeTe) through first-principles calculations. Our results show that when TMD monolayers are stacked vertically with Janus monolayers, an intrinsic electric field appears because of the lack of mirror symmetry and charge accumulation, which leads to the origin of versatile properties in Janus vdWHs. In addition, the interlayer twist also induces an electronic phase crossover from indirect to direct bandgap at some specific rotation angles. It is interesting to note that the Janus $\hbox {MoS}_2$/MoSeTe ($\theta$ = 19.11$^{\circ }$, 40.89$^{\circ }$), $\hbox {WS}_2$/WSeTe ($\theta$ = 19.11$^{\circ }$, 30$^{\circ }$), $\hbox {MoS}_2$/WSeTe ($\theta$ = 19.11$^{\circ }$), $\hbox {WSe}_2$/MoSeTe ($\theta$ = 16.10$^{\circ }$), $\hbox {WS}_2$/MoSeTe ($\theta$ = 40.89$^{\circ }$) vdWHs have direct bandgap with type-II band alignment, which is an essential parameter for photocatalytic water splitting applications. The interfacial interaction also induces high carrier mobility, efficient charge separation and high value of refractive index. Furthermore, the Janus vdWHs exhibit dramatically reduced direct band gaps and more efficient optical absorption from the UV to visible light range. The enhancement rate of absorption coefficient of the Janus vdWHs at $\theta$ = 0$^{\circ }$) is quite high ($\sim$ 10$^5$ $\hbox {cm}^{-1}$) in the visible region due to the perfect interlayer coupling between the monolayers. Our findings not only provide a compelling platform for exploring the behavior of the Janus vdWHs but also provides theoretical guidance for the designing of novel 2D nanodevices.

### Supplementary Information

Supplementary Information.

## Data Availability

The data that support the findings of this study are available from the corresponding author upon reasonable request.
