# Supplementary material for: Twist-assisted optoelectronic phase control in two-dimensional (2D) Janus heterostructures
Source: Sci Rep. 2023 Aug 22;13:13696. doi: 10.1038/s41598-023-39993-8 (PMC10444812; doi:10.1038/s41598-023-39993-8)
Supplement: Supplementary file 1 — Supplementary Information. [file 41598_2023_39993_MOESM1_ESM.pdf]

Supporting Information

Twist-assisted Optoelectronic Phase control in Two-dimensional (2D) Janus heterostructures

S. Kar[1], P. Kumari[1], M. V. Kamalakar[2], S. J. Ray[1*]

[1]*Department of Physics, Indian Institute of Technology Patna, Bihta 801106, India*

[2]*Department of Physics and Astronomy, Uppsala University, Box 516, SE-75120 Uppsala, Sweden*

*Electronic address: ray@iitp.ac.in

TABLE S1: Bandgap and lattice parameters of monolayers.

Material	a = b (Å)	E_g (eV) (PBE)	E_g (eV) (HSE)	Type
MoS_2	3.1604	1.68	2.271	Direct
$MoSe_2$	3.288	1.43	1.980	Direct
WS_2	3.1532	1.95	2.442	Direct
WSe_2	3.282	1.64	2.148	Direct
MoSeTe	3.4	1.14	2.17207	Indirect
WSeTe	3.405	1.41	2.4505	Indirect

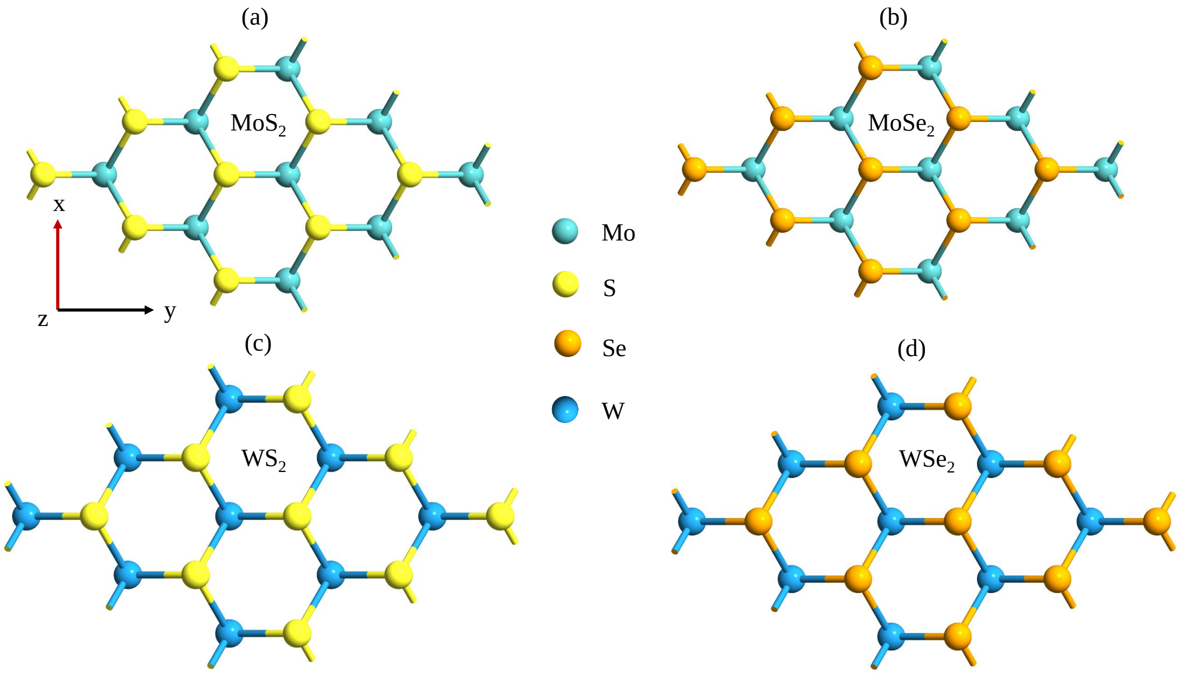

FIG. S1: The optimised geometric structures of (a) MoS_2, (b) $MoSe_2$, (c) WS_2, and (d) WSe_2.

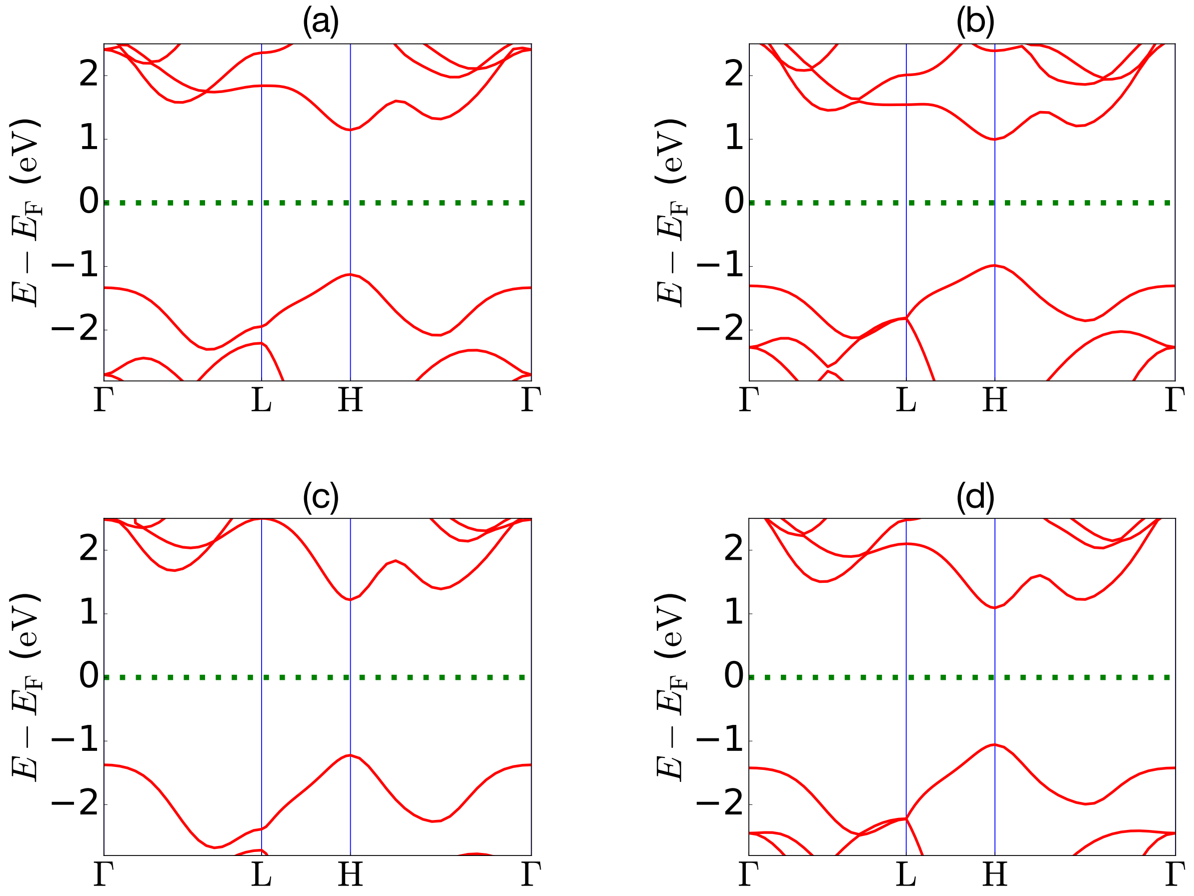

FIG. S2: The band diagram of: (a) MoS_2, (b) $MoSe_2$, (c) WS_2, and (d) WSe_2; by using HSE calculation.

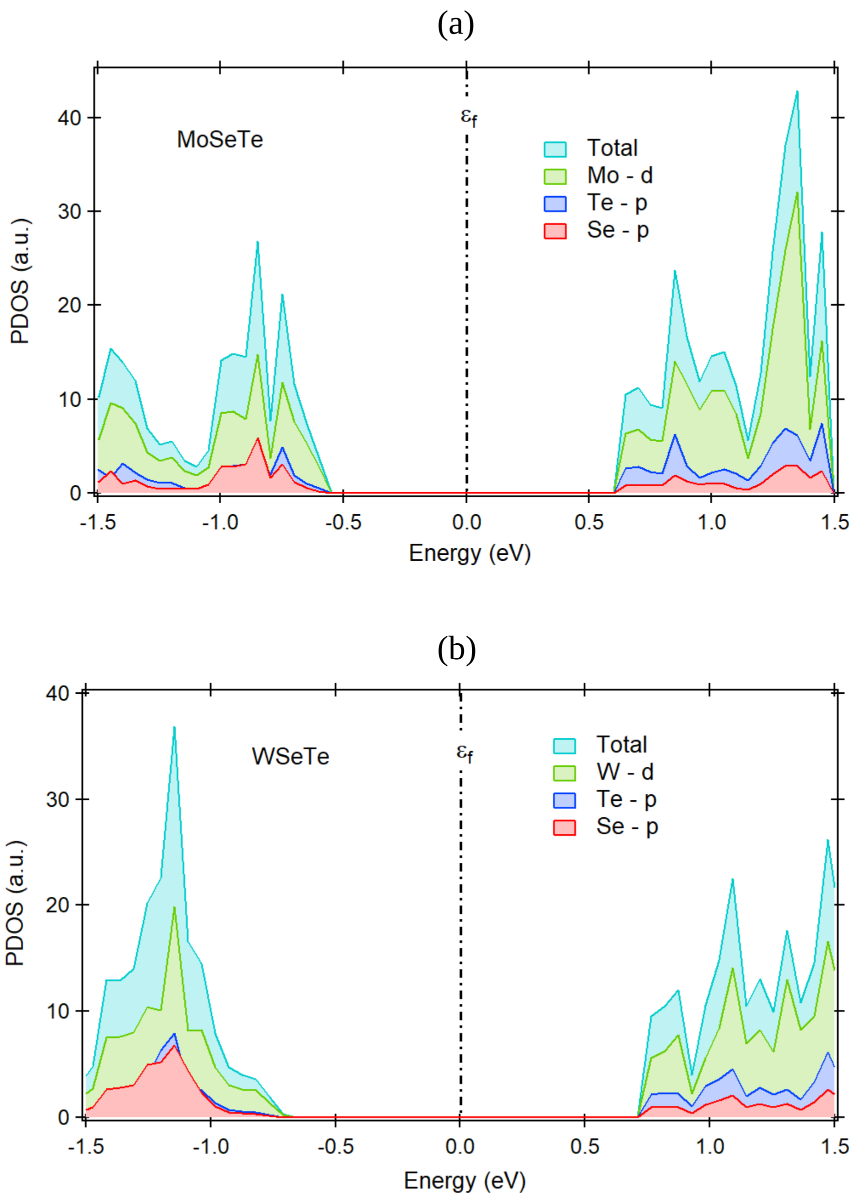

FIG. S3: The projected density of states (PDOS) of Janus (a) MoSeTe and (b) WSeTe monolayer.

TABLE S2: Rotation angle (θ), Number of atoms (N), Lattice mismatch (LM), Interlayer distance (d) XS_2/XSeTe heterostructure (X = Mo, W).

Angle(^{0})	N	MoS_2/MoSeTe		MoS_2/WSeTe		WS_2/MoSeTe		WS_2/WSeTe	
		LM(%)	d(Å)	LM(%)	d(Å)	LM(%)	d(Å)	LM(%)	d(Å)
0	54	2.43	3.8	2.51	3.21	2.43	3.81	2.56	3.2
19.11	48	1.75	3.61	1.68	3.41	1.75	3.61	1.63	3.41
30	42	2.36	3.63	2.28	3.45	2.36	3.63	2.23	3.44
40.89	49	1.79	3.65	1.70	3.43	1.80	3.63	1.67	3.43

TABLE S3: Rotation angle (θ), Number of atoms (N), Lattice mismatch (LM), Interlayer distance (d) XSe_2/XSeTe heterostructure (X = Mo, W).

Angle(^{0})	N	MoSe_2/MoSeTe		MoSe_2/WSeTe		WSe_2/MoSeTe		WSe_2/WSeTe	
		LM(%)	d(Å)	LM(%)	d(Å)	LM(%)	d(Å)	LM(%)	d(Å)
0	54	1.12	4.0	1.17	3.2	1.10	3.81	1.15	3.2
16.10	75	0.22	3.61	0.17	3.6	0.24	3.6	0.19	3.22
21.79	42	1.15	3.63	1.15	3.8	1.14	3.8	1.07	3.6
38.21	49	1.19	3.65	1.1	3.6	1.1	3.63	1.67	3.64
43.90	77	0.25	3.6	0.19	3.6	0.29	3.6	0.15	3.4

TABLE S4: Rotation angle (θ), Number of atoms (N), Lattice mismatch (LM), Interlayer distance of Janus MoSeTe/WSeTe heterostructure.

Angle(^{0})	N	LM(%)	d(Å)
0	54	0.05	3.2
16.10	75	1.30	3.8
21.79	78	0.04	3.6
38.21	42	0.06	3.62
43.90	71	1.32	3.61

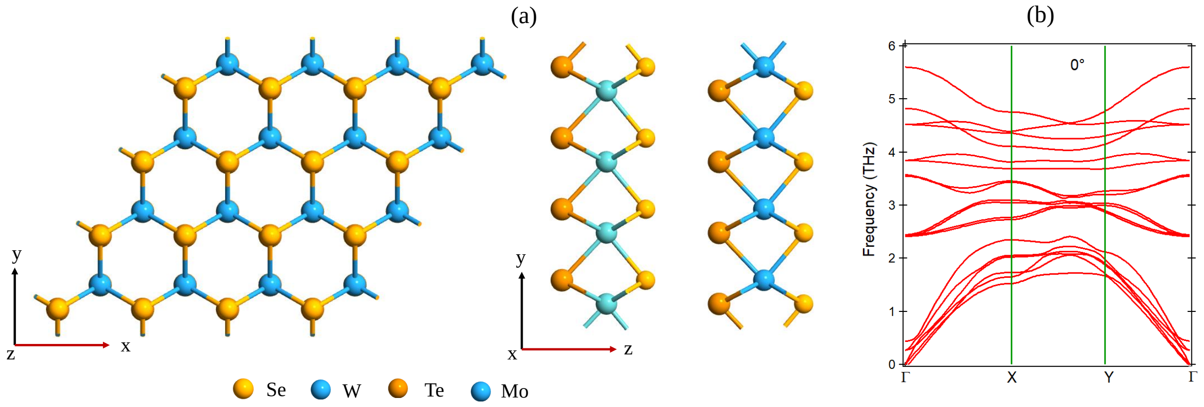

FIG. S4: (a) Top and side view and (b) phonon bandstructure of the Janus MoSeTe/WSeTe heterostructure at $\theta = 0^0$.

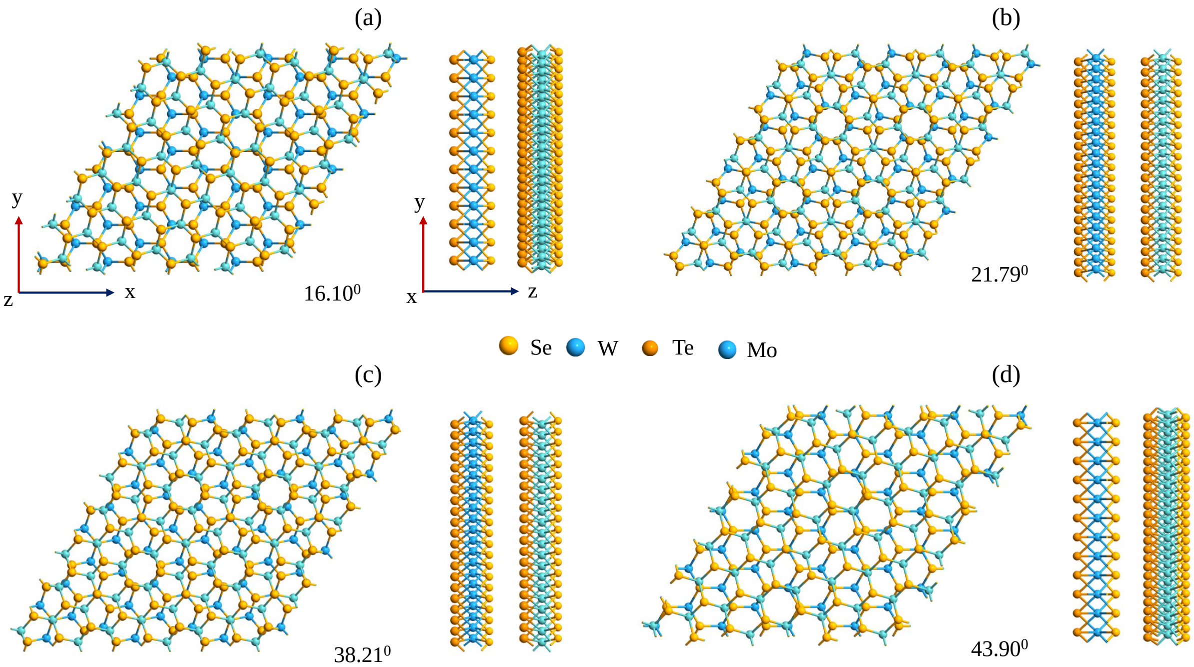

FIG. S5: Top and side view of the Janus MoSeTe/WSeTe heterostructure at different rotation angles: (a)16.10^0, (b)21.79^0, (c) 38.21^0, and (d) 43.90^0.

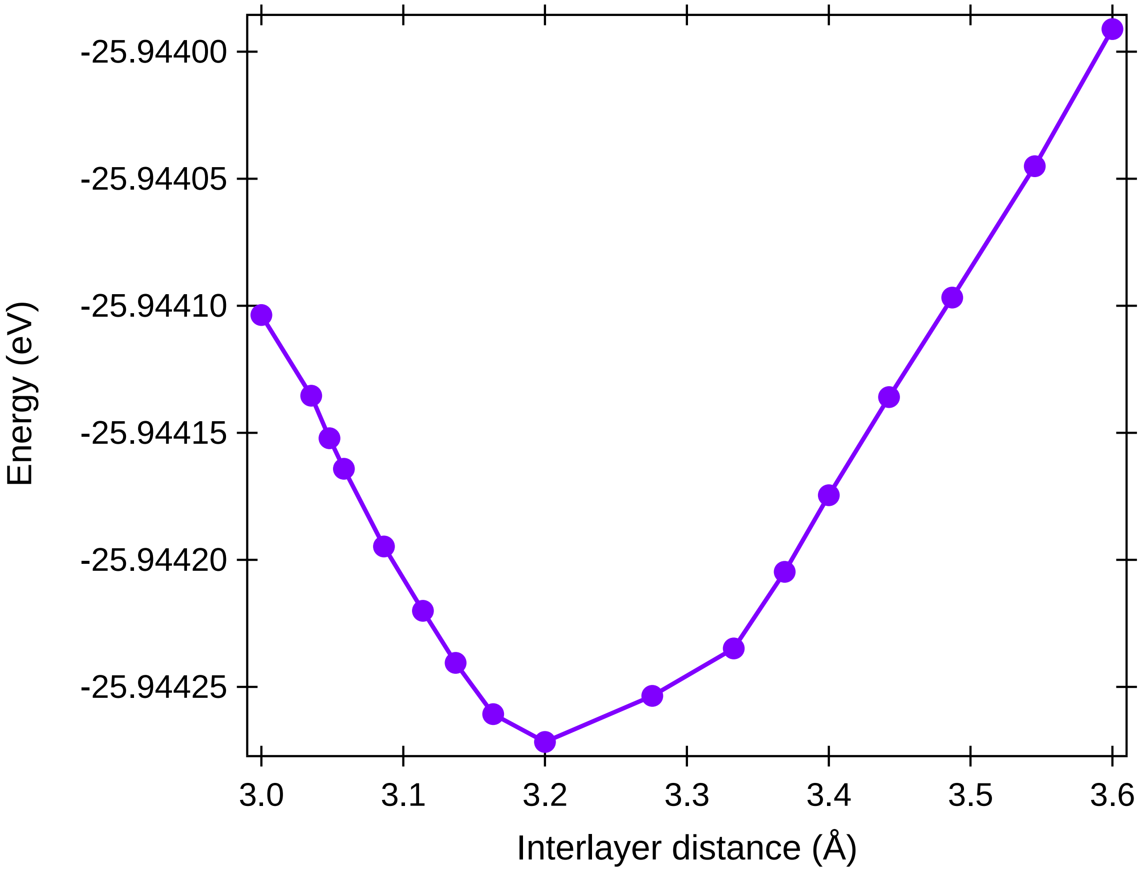

FIG. S6: The calculated interlayer distance (d) for Janus WSe_2/WSeTe heterostructure at $\theta = 0^0$.

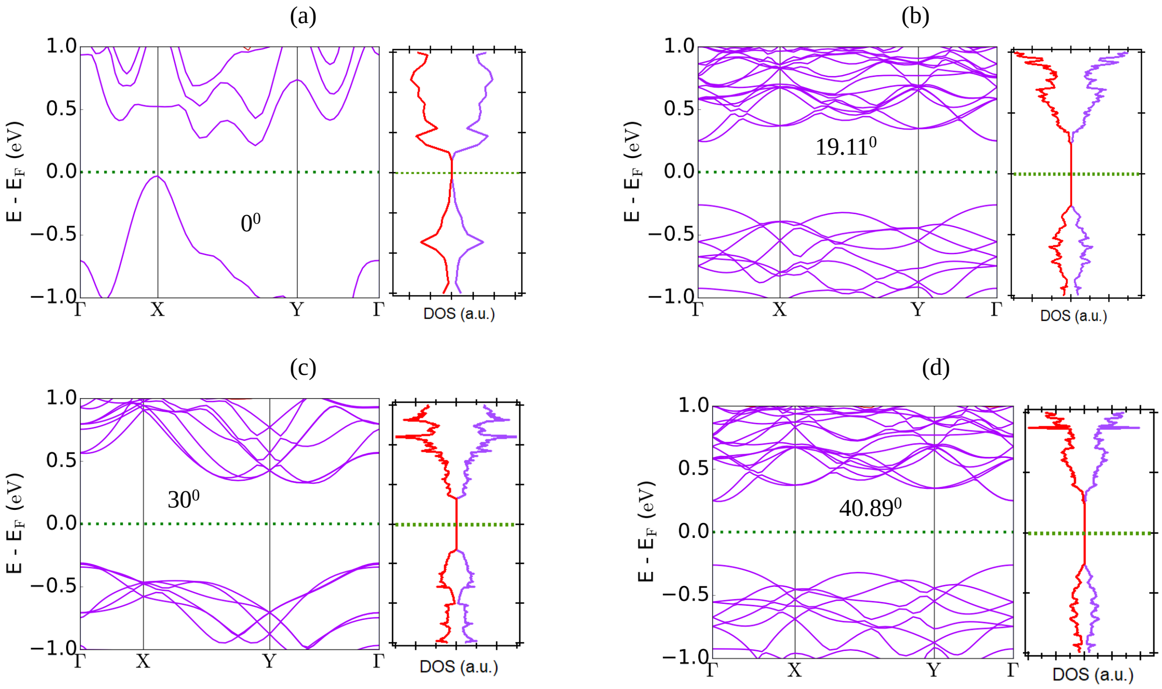

FIG. S7: Bandstructure and density of states (DOS) of Janus MoS_2/MoSeTe heterostructure at different rotation angles: (a) 0^0, (b) 19.11^0, (c) 30^0, and (d) 40.89^0.

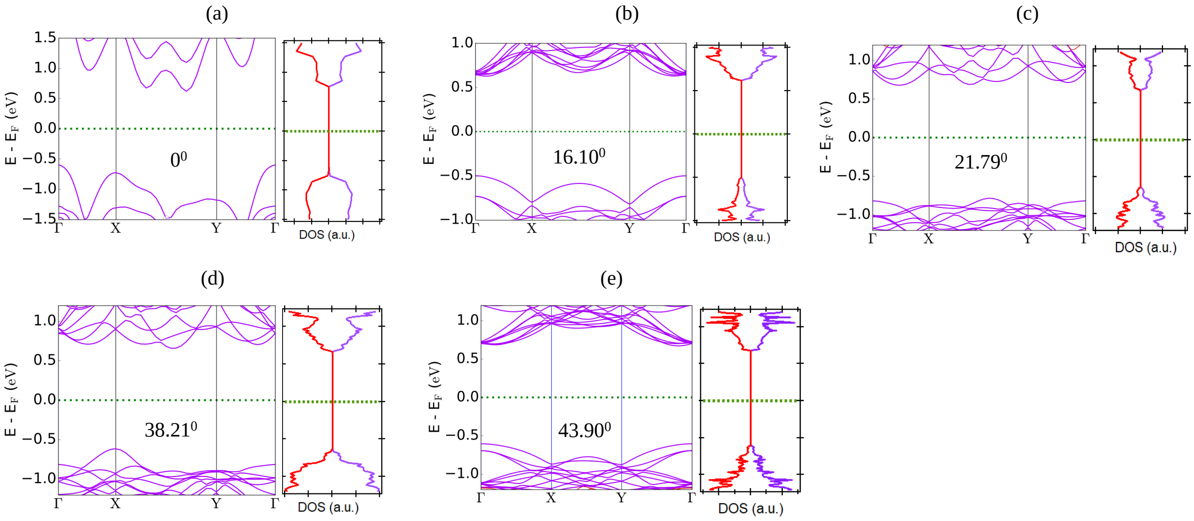

FIG. S8: Bandstructure and density of states (DOS) of Janus WSe_2/WSeTe heterostructure at different rotation angles: (a) 0^0, (b) 16.10^0, (c) 21.79^0, (d) 38.21^0, and (e) 43.90^0.

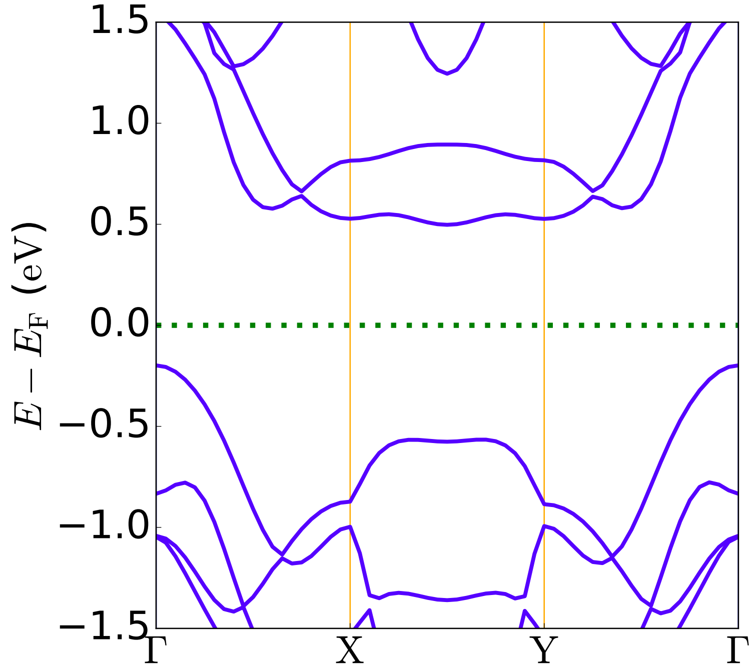

FIG. S9: The band structure of MoSeTe/WSeTe heterostructure at $\theta = 0^0$ with vdW and dipole corrections.

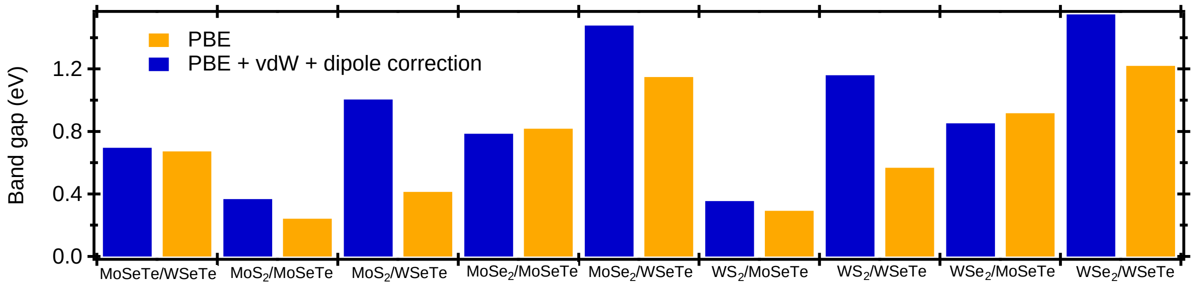

FIG. S10: The variation of the bandgap of the Janus vdW Heterostructures at $\theta = 0^0$.

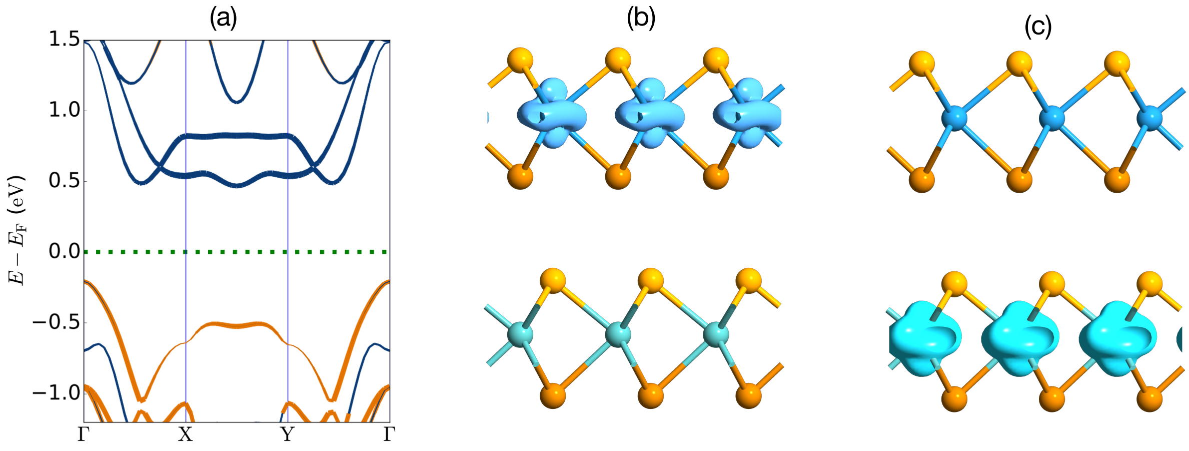

FIG. S11: (a)The projected band structure of MoSeTe/WSeTe at $\theta = 0^0$. The partial charge density of MoSeTe/ WSeTe at $\theta = 0^0$: (b)Valence band region, (c)Conduction band region.

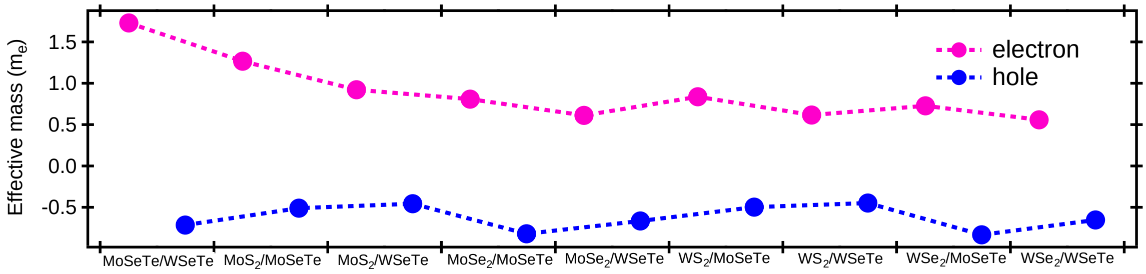

FIG. S12: The effective mass of Janus vdW Heterostructures at $\theta = 0^0$.

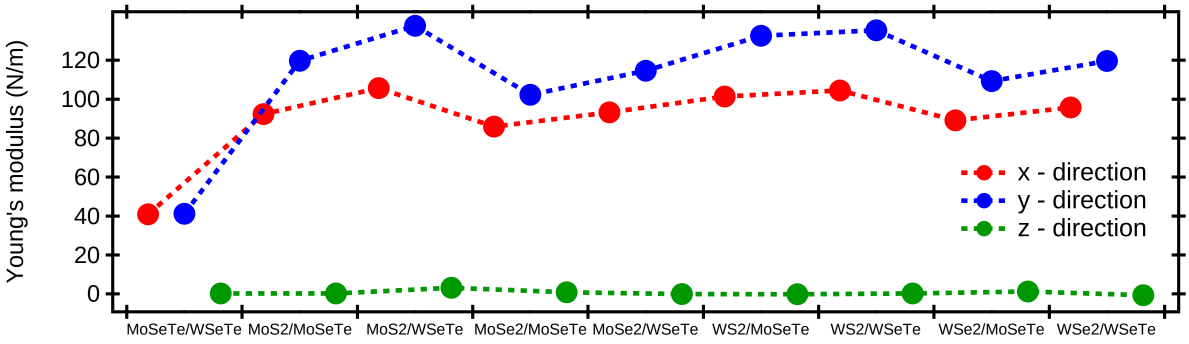

FIG. S13: The Young modulus of Janus vdW heterostructure at $\theta = 0^0$.

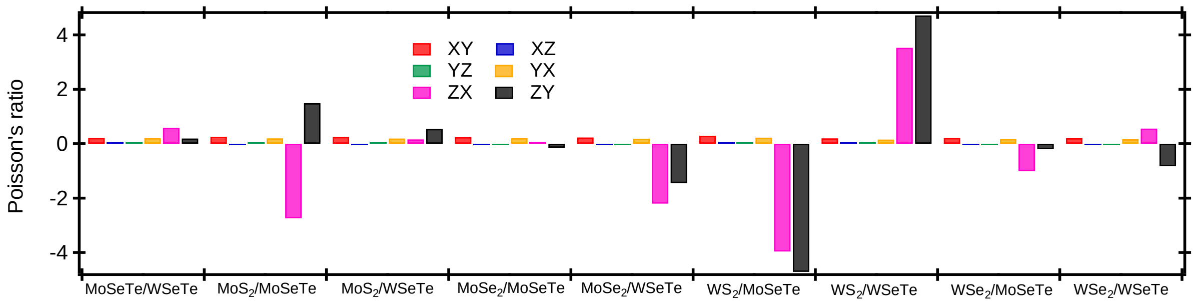

FIG. S14: Figure R6: The Poisson's ratio of Janus vdW heterostructure at $\theta = 0^0$.

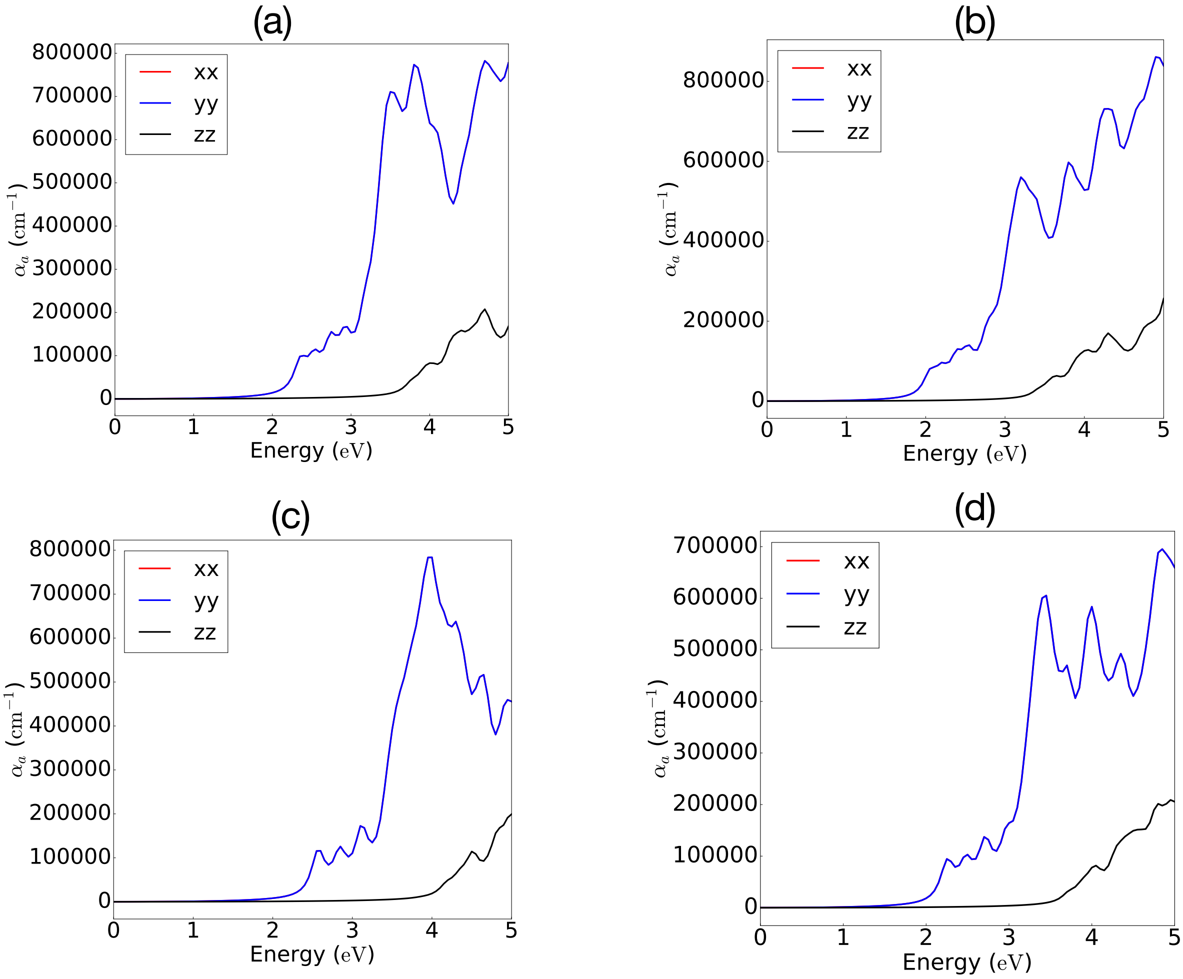

FIG. S15: The absorption coefficient of: (a)MoS_2, (b)$MoSe_2$, (c)WS_2, and (d)WSe_2; by using HSE calculation.

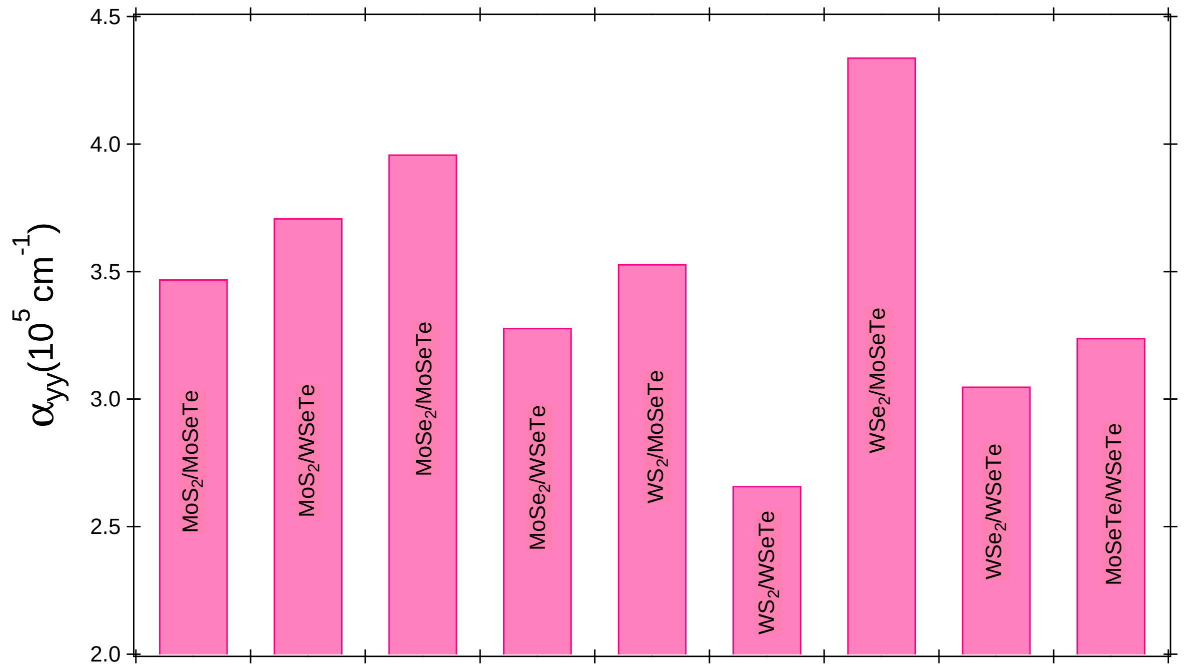

FIG. S16: The variation of absorption (photon energy = 3 eV) coefficient at $\theta = 0^0$.
